# Effect of switching administration of alendronate after teriparatide for the prevention of BMD loss around the implant after total hip arthroplasty, 2-year follow-up: a randomized controlled trial

**DOI:** 10.1186/s13018-020-1547-5

**Published:** 2020-01-16

**Authors:** Akira Morita, Naomi Kobayashi, Hyonmin Choe, Hiroyuki Ike, Taro Tezuka, Shota Higashihira, Yutaka Inaba

**Affiliations:** 1 Department of Orthopaedic Surgery, Yokohama City University, 3-9 Fukuura, Kanazawa-ku, Yokohama 236-0004 Japan; 2 Department of Orthopaedic Surgery, Yokohama City University Medical Center, 4-57 Urafune-cho, Minami-ku, Yokohama, 232-0024 Japan

**Keywords:** Bone mineral density (BMD), Total hip arthroplasty (THA), Teriparatide, Alendronate (ALD)

## Abstract

**Background:**

Stress shielding after total hip arthroplasty (THA) can induce bone mineral density (BMD) loss around the femoral implant. Several studies using drug have described methods to prevent BMD loss around implants following THA. Switching from teriparatide to alendronate was reported to increase lumbar BMD; on the other hands, it is unclear whether switching from teriparatide to alendronate is effective around the implant. The aim of this study is that changes in BMD is compared in patients switched from teriparatide to alendronate, in patients treated with alendronate alone, and in control patients without medication after total hip arthroplasty.

**Patients and methods:**

Patients were randomized into three groups, those switched to alendronate after teriparatide (switch: *n* = 17), those receiving continuous alendronate (ALD: *n* = 15), and control untreated patients (control: *n* = 16) and followed up for 2 years after THA. Baseline periprosthetic BMD was measured by dual-energy X-ray absorptiometry (DEXA) 1 week after THA, followed by subsequent measurements at 1 and 2 years postoperatively. Lumbar BMD was also evaluated at preoperatively, 1 and 2 years postoperatively.

**Results:**

Two years after surgery, BMD (%) at zone 1 was significantly higher in the switch group than in the control group (*P* = 0.02). BMD (%) at zone 7 was significantly higher in the switch and ALD groups than in the control group (*P* = 0.01, *P* = 0.03). Lumbar BMD (%) anterior-posterior (AP) side was significantly higher in the switch group than in the ALD and control groups 2 years after surgery. On the other hand, lumbar BMD (%) lateral side was significantly higher in the switch and ALD groups than control group 2 years after surgery.

**Conclusions:**

Switching therapy had a significant effect on BMD of the lumbar spine and zones 1 and 7 at 2 years postoperatively. At zone 1 in particular, it was found to be more effective than ALD alone.

**Trial registration:**

UMIN, registry number UMIN000016158. Registered 8 January 2015

## Introduction

Total hip arthroplasty (THA) is a promising surgical treatment, providing pain relief, functional recovery, and long-term stable clinical results, with 20-year implant survival rates higher than 90% [1, 2]. However, stress shielding can induce bone mineral density (BMD) loss around the femoral implant [3–5]. Although BMD loss around the implant has not been found to result in poorer clinical outcomes after THA, a large retrospective cohort study suggested that the use of bisphosphonate decreased the risk of revision after THA [6]. In addition, BMD loss around the implant may be associated with an increased risk of periprosthetic fractures [7]. Indeed, many periprosthetic fractures have been associated with decreased periprosthetic BMD [8]. Thus, prevention of BMD loss after THA seems to be clinically desirable.

Several recent studies have described methods to prevent BMD loss around implants following THA [9–11]. Teriparatide and alendronate, which have adverse effects on bone metabolism, are principal drugs for treating osteoporosis. A randomized controlled trial comparing teriparatide with alendronate for the prevention of BMD loss around the implant over 1 year [12] found that both significantly prevented BMD loss, particularly in the medial proximal femur. Although combination therapy with teriparatide and alendronate was not effective for osteoporosis [13], switching from teriparatide to alendronate was reported to increase lumbar BMD [14, 15]. These findings suggested that switching from teriparatide to alendronate may also have a positive effect on the BMD around the implant after THA.

The primary goal of this study was therefore to compare changes in BMD in three randomly assigned groups, patients switched from teriparatide to alendronate, patients treated with alendronate alone, and a control untreated group, for 2 years after THA.

## Patients and methods

**Fig. 1:**
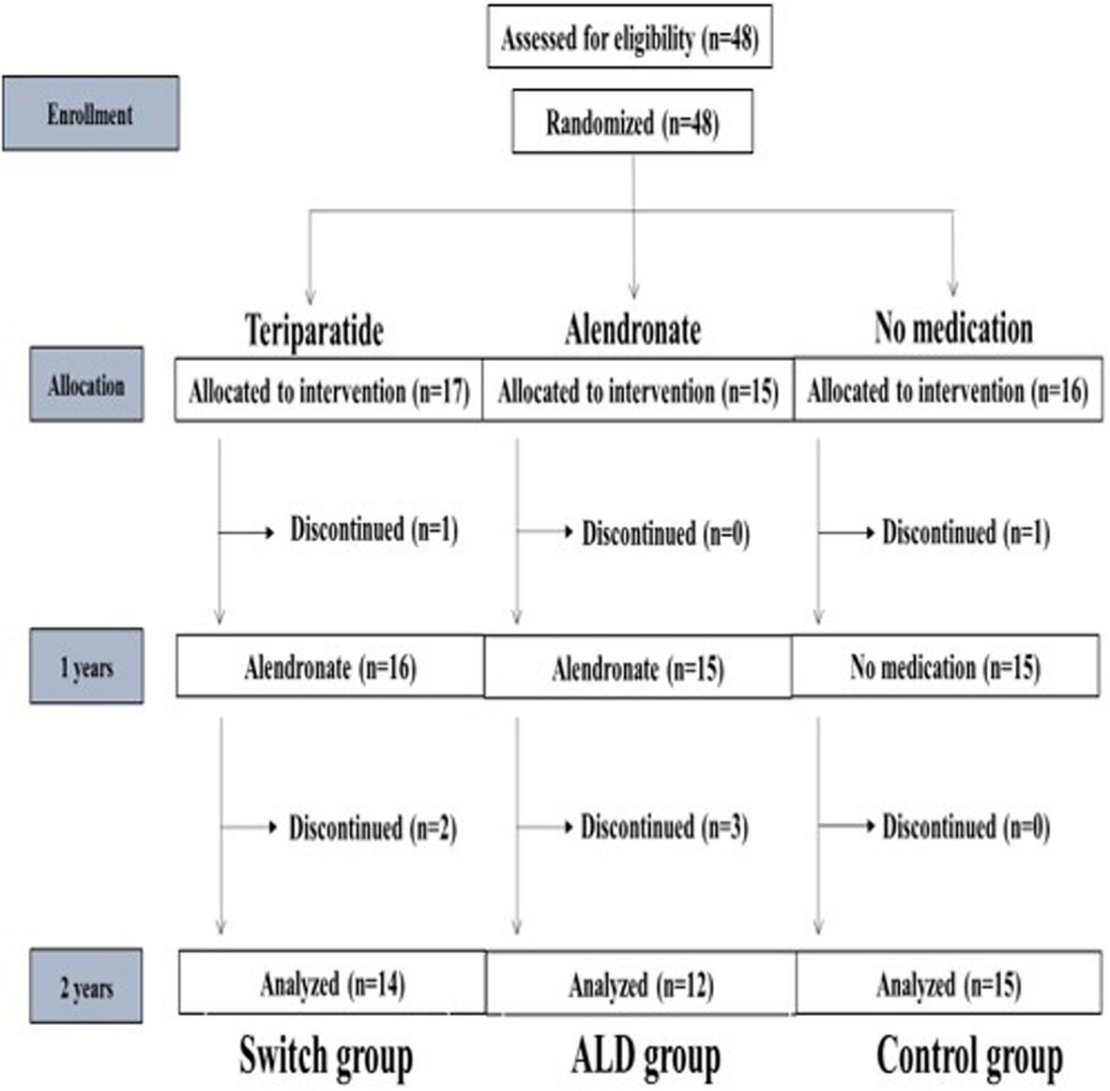
Diagram showing patient randomization

This randomized controlled trial (RCT) was an extension of a previous reported study registered in the University Hospital Medical Information Network (UMIN) clinical trial registry (registry number: UMIN000016158) ^9^. Forty-eight subjects, 42 women, and 6 men, were included, all of whom provided written informed consent and underwent primary cementless THA in our hospital from December 2011 to September 2013. Their mean (± standard deviation) age at the time of surgery was 65 ± 10 years, and their mean body mass index (BMI) was 23.4 ± 3.9 kg/m^2^. All patients were implanted with the same cementless femoral component (SL-PLUS MIA, Smith and Nephew, Inc. Memphis, TN), cementless acetabular component (REFLECTION, Smith and Nephew, Inc.), and cross-linked polyethylene liner (XLPE liner, Smith and Nephew, Inc.). Patients were excluded if they had (1) a history of disorder that might affect the bone or mineral metabolism, (2) gastrointestinal disease, (3) renal dysfunction, or (4) previously taken estrogen, progesterone, androgens, calcitonin, glucocorticoid, bisphosphonate, calcium supplements, or vitamin D. Figure 1 shows the study flow diagram. Of the 48 patients, 17 were randomized to the switch group, 15 to the ALD group, and 16 to the control group (Fig. 1). Patients in the switch group were started on daily injections of 20 μg/day teriparatide (Forteo, Eli Lilly Japan K.K.), beginning 2 weeks after THA and continuing for 1 year. These patients were subsequently switched to oral administration of ALD (35 mg/week) continuously for 1 year. Patients in the ALD group were started on oral administration of alendronate (35 mg/week; Bonalon, Teijin Pharma Limited) beginning 2 weeks after THA and continuing for 2 years. Patients in the control group received no drug for 2 years. During the observation period, 7 patients discontinued the trial, four (two in the switch and two in the ALD groups) due to discontinued drug use, two (one in the switch and one in the ALD groups) for discontinued follow-up, and one in the control group due to THA in the contralateral hip 24 weeks after the primary THA. Patient activity levels and clinical outcomes were evaluated by measuring the University of California, Los Angeles (UCLA), activity score, Japanese Orthopaedic Association (JOA) hip score, and Harris Hip Score (HHS) preoperatively and after 1 and 2 years. All patients started to use a wheelchair on the first postoperative day, commencing gait exercises with full-weight bearing as soon as possible.

**Fig. 2:**
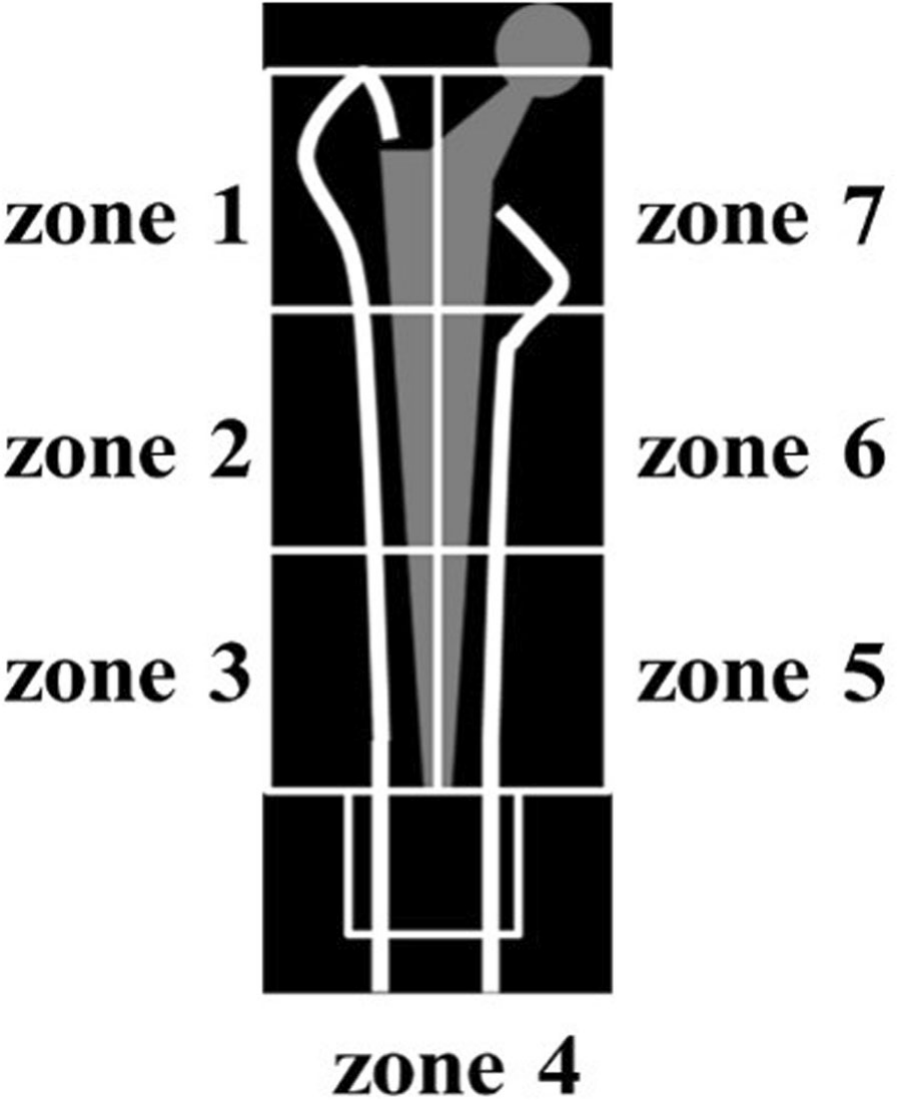
Gruen’s zone classification

Baseline periprosthetic BMD was measured 1 week after THA, followed by subsequent measurements at 1 and 2 years using dual-energy X-ray absorptiometry (DEXA) (QDR 2000, Hologic, Waltham, MA). The periprosthetic zones described by Gruen were used for the regions of interest (ROIs) (Fig. 2). Baseline lumbar BMD was also measured preoperatively, followed by subsequent measurements at 1 and 2 years postoperatively for the L2 to L4 lumbar anterior-posterior (AP) direction and lateral direction using DEXA. The rate of change (%) of BMD relative to baseline was statistically evaluated using one-way analysis of variance and Tukey-Kramer method. All statistical analyses were performed using EZR software [16], which modified version of R commander designed to add statistical functions frequently used in biostatistics. A *p* value < 0.05 was considered statistically significant.

## Results

**Fig. 3:**
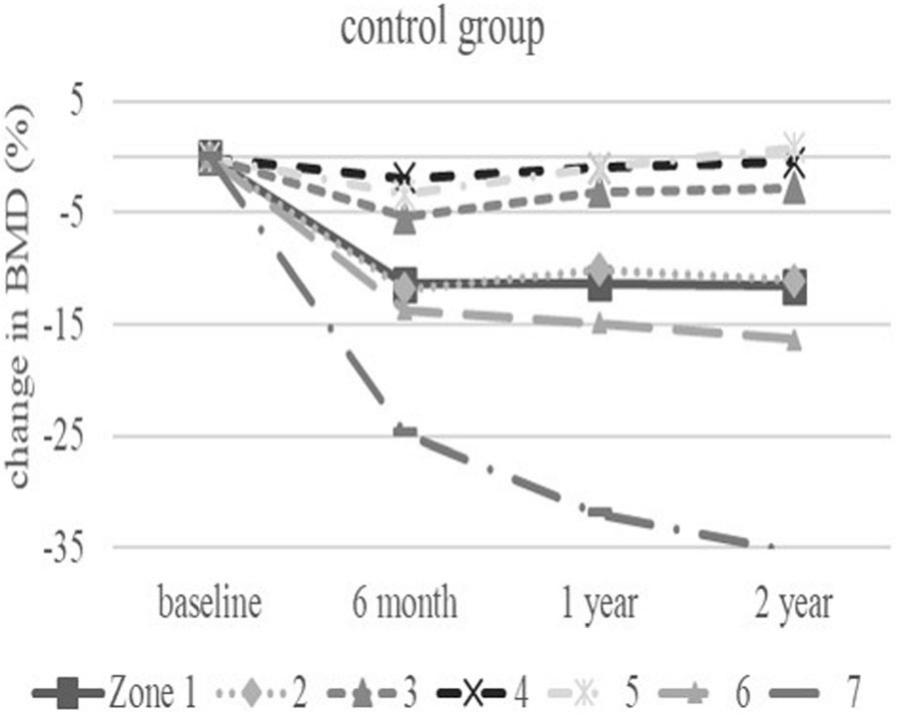
Change in BMD (%) from baseline in each zone of the control group over 2 years

**Fig. 4:**
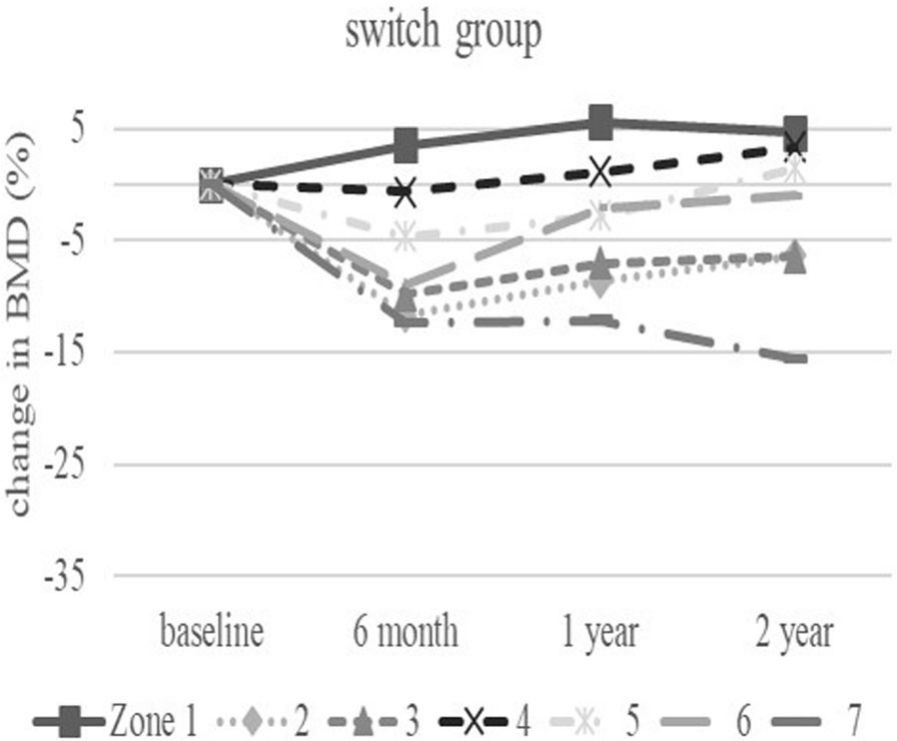
Change in BMD (%) from baseline in each zone of the switch group over 2 years

**Fig. 5:**
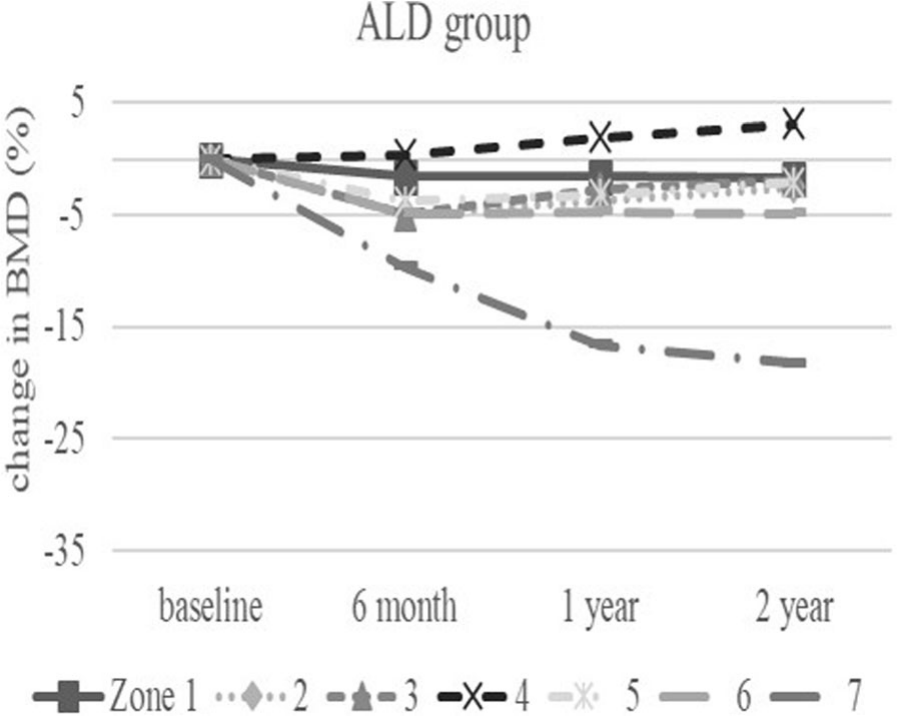
Change in BMD (%) from baseline in each zone of the ALD group over 2 years

**Fig. 6:**
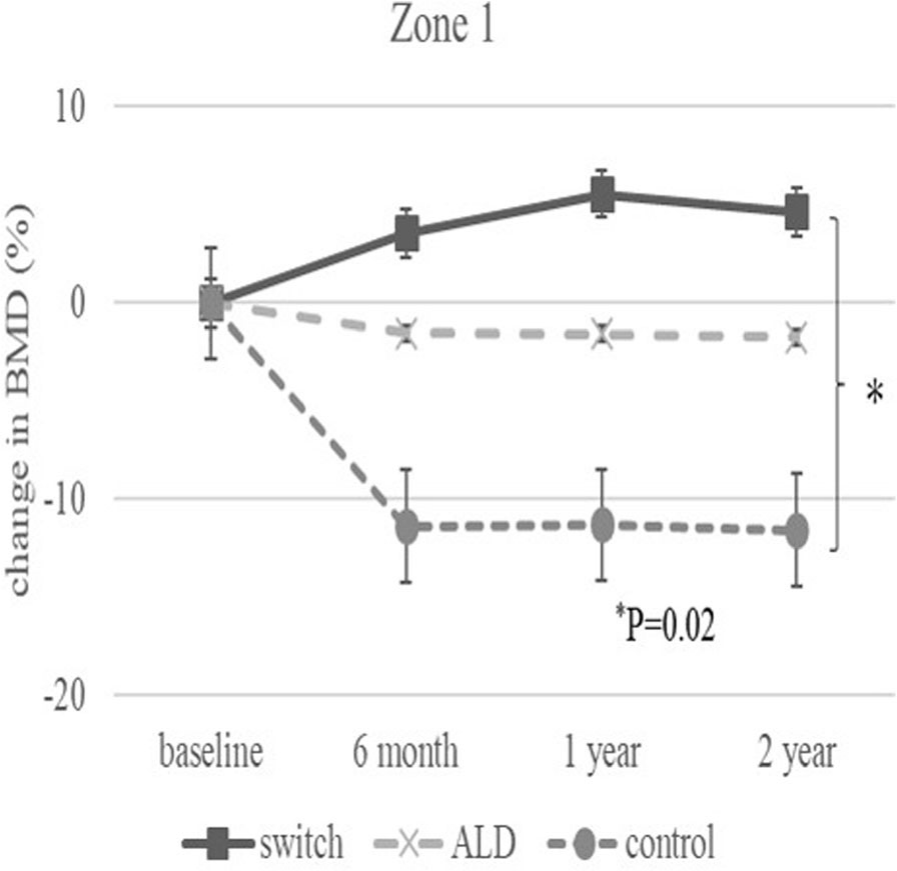
Change in BMD (%) from baseline in the three patient groups at zone 1 over 2 years. Significant differences were observed between the switch and control groups at 2 years (*P* = 0.02)

**Fig. 7:**
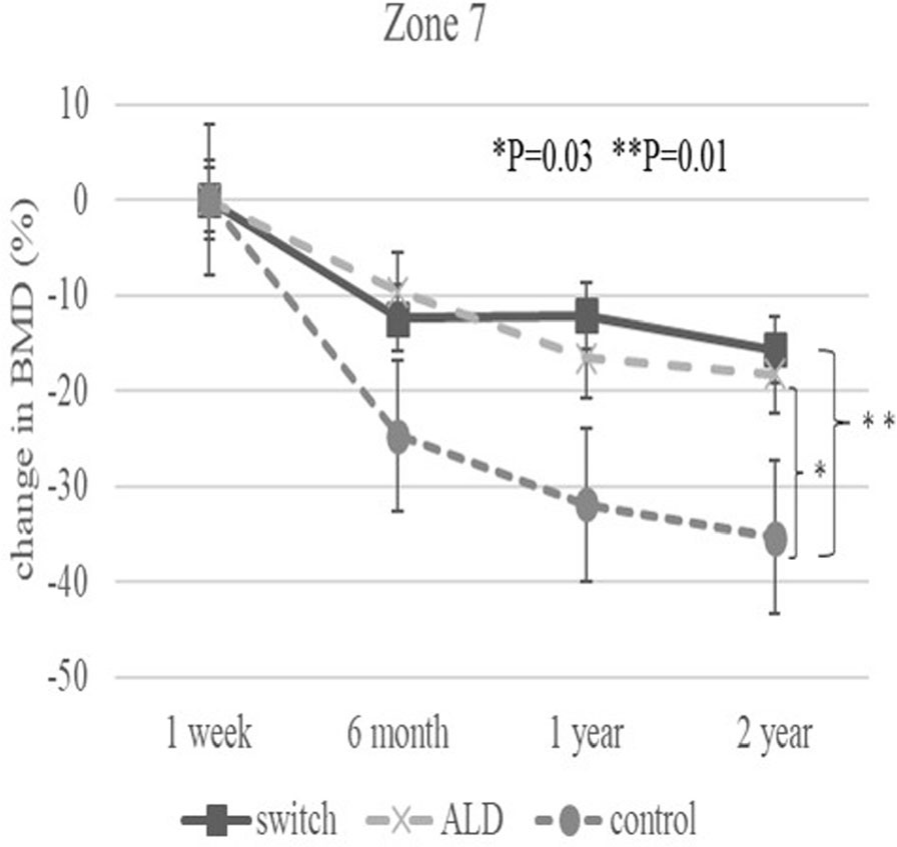
Change in BMD (%) from baseline in the three patient groups at zone 7 over 2 years. Significant differences were observed between the switch and control groups (*P* = 0.01) and between ALD and control groups at 2 years (*P* = 0.03)

**Fig. 8:**
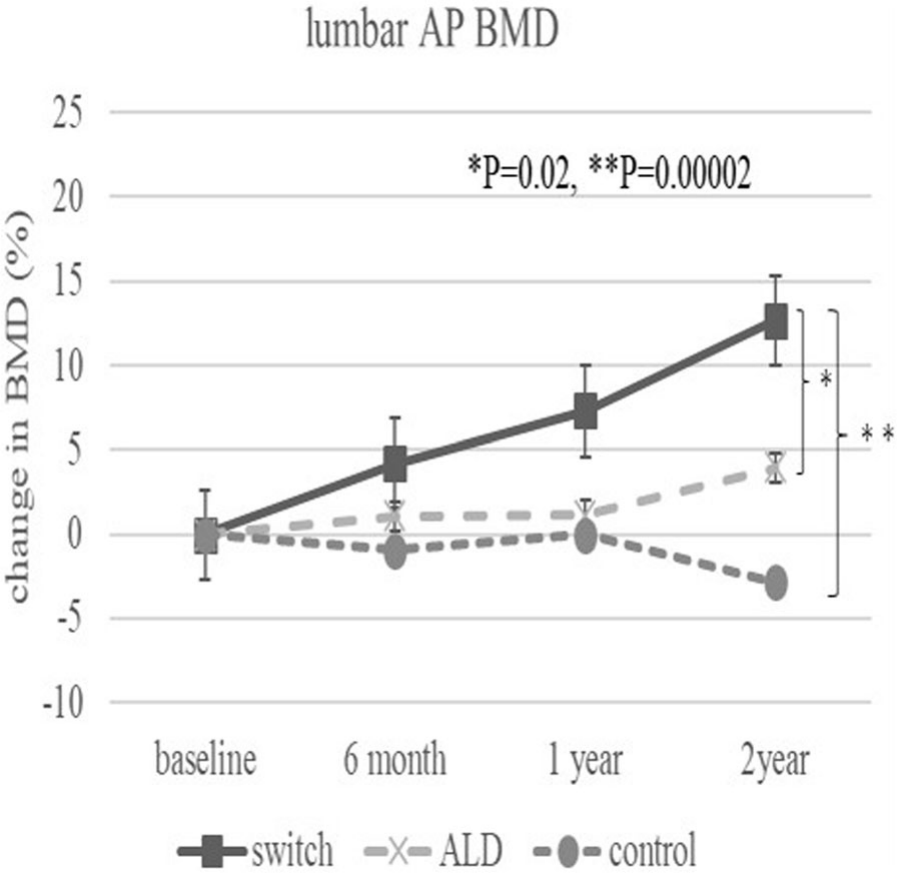
Change in BMD (%) from baseline in the three patient groups at the lumbar spine BMD AP side over 2 years. Significant differences were observed between the switch and ALD groups (*P* = 0.02) and between the switch and control groups (*P* = 0.00002)

**Fig. 9:**
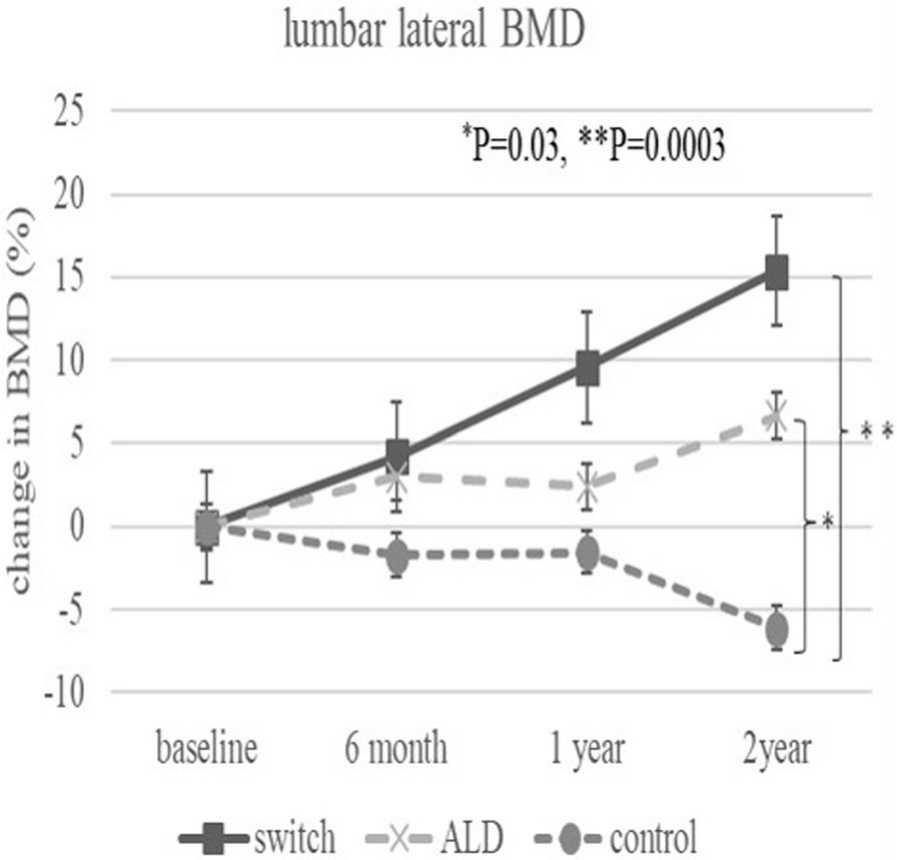
Change in BMD (%) from baseline in the three patient groups at the lumbar spine BMD lateral side over 2 years. Significant differences were observed between the switch and control groups (*P* = 0.0003) and between the ALD and control groups (*P* = 0.03)

The baseline demographic characteristics and preoperative activity and functional score in each group are shown in Table 1. There were no significant differences in any factor among these groups. Table 2 shows activity and functional score 2 years after surgery in each group. There were no significant differences in any of these scores. Figure 3 shows the rate of BMD change (%) of each zone in the control group up to 2 years after surgery. Relative to baseline, the rates of BMD change (%) at 2 years after surgery were − 11.6 ± 13.1% at zone 1, − 11.0 ± 15.5% at zone 2, − 2.7 ± 6.3% at zone 3, − 0.4 ± 6.4% at zone 4, + 0.8 ± 8.3% at zone 5, − 16.3 ± 8.6% at zone 6, and − 35.4 ± 10.7% at zone 7. The BMD changes (%) from baseline to 2 years differed significantly at zone 1 and 7 (*P* < 0.01 each). Figure 4 shows the rates of BMD changes in the switch group. Relative to baseline, the rates of BMD change (%) at 2 years after surgery were + 4.7 ± 13.6% at zone 1, − 6.4 ± 13.0% at zone 2, − 6.5 ± 8.2% at zone 3, + 3.4 ± 4.5% at zone 4, + 1.5 ± 5.3% at zone 5, − 1.0 ± 22.7% at zone 6, and − 15.7 ± 15.6% at zone 7. Figure 5 shows the rates of BMD changes in the ALD group. Relative to baseline, the rates of BMD change (%) at 2 years after surgery were + 1.7 ± 18.2% at zone 1, − 2.5 ± 14.2% at zone 2, − 1.9 ± 7.3% at zone 3, + 3.1 ± 4.2% at zone 4, − 2.1 ± 10.0% at zone 5, − 4.9 ± 16.7% at zone 6, and − 18.2 ± 22.5% at zone 7. Figure 6 shows the differences in BMD changes at zone 1 in the three groups. There was a significant difference between the switch and control groups after 2 years postoperatively (*P* = 0.02), but no significant difference between the ADL and control groups. Figure 7 shows BMD changes in the three groups at zone 7. There was a significant difference between switch, ALD, and control groups; however, no significant difference between switch and ALD groups. Figure 8 shows that lumbar BMD AP side changes at 2 years were + 12.7 ± 7.1% in the switch group, + 4.0 ± 6.1% in the ALD group, and − 2.8 ± 5.8% in the control group, with significant differences between switch and ALD groups (*P* = 0.02) and switch and control (*P* = 0.00002). Figure 9 shows that the lumbar BMD lateral side changes at 2 years were + 15.4 ± 11.7% in the switch group, + 6.7 ± 8.4% in the ALD group, and − 6.1 ± 11.2% in the control group. There was a significant difference between switch, ALD, and control groups, but no significant difference between switch and ALD groups. There were no significant differences in BMD changes among the three groups at zones 2, 3, 4, 5, and 6.

**Table 1:** Baseline demographic and clinical characteristics of the three patient groups

	Switch	ALD	Control	*P* value
Number of subjects	17	15	16	
Male/female	1/16	1/14	4/11	
Average age, years	65.8 ± 10.6	63.8 ± 9.3	64.8 ± 9.2	N.S.
Average BMI, kg/m^2^	21.9 ± 4.2	24.0 ± 2.6	24.6 ± 3.8	N.S.
JOA score	49.8 ± 9.7	53.3 ± 14.5	48.6 ± 11.0	N.S.
UCLA activity score	4.4 ± 1.2	5.0 ± 0.9	4.4 ± 1.4	N.S.
HHS	56.9 ± 9.9	54.7 ± 12.5	49.2 ± 10.6	N.S.
Lumbar spine BMD, g/cm^2^	0.8 ± 0.2	0.9 ± 0.1	0.9 ± 0.1	N.S.

**Table 2:** Clinical function scores of the three patient groups 2 years after surgery

	Switch group	ALD group	Control group	*P* value
JOA score	92.7 ± 7.1	97.7 ± 2.9	94.8 ± 5.7	N.S.
UCLA activity score	6.5 ± 0.7	6.1 ± 0.6	6.4 ± 1.0	N.S.
HHS	96.8 ± 4.1	98.5 ± 2.0	96.7 ± 4.1	N.S.

## Discussion

This RCT assessed the effects of three treatments on BMD around the implant and lumbar spine BMD for 2 years after THA. This study confirmed that switching from teriparatide to alendronate had significant effects in zones 1 and 7 and on lumbar BMD.

BMD loss around the femoral implant has been observed in several previous studies [17, 18]. Although many factors are involved, environmental changes mainly caused by mechanical stress are likely most important. Differences in stem design, such as between the Zweymuller and fit-and-fill types, directly influence the mechanical property of the femur, thereby affecting the BMD as a result [4]. Particularly in the proximal medial region, i.e., zone 7, the correlation between mechanical stress and BMD is significant [5]. By contrast, the shape of the proximal femur, such as stove pipe or champagne flute, is also important for mechanical stress and BMD even when using the same implant type [19]. Thus, BMD loss around the implant likely involves an inter-relationship between implant and femoral configuration. It is not realistic to change such mechanical configuration after surgery, making drug intervention to prevent BMD less practical.

Several studies have tested the ability of bisphosphonate to prevent BMD loss around the femoral implant [10, 11, 20–24], whereas there is no clear evidence of poorer clinical outcomes due to BMD loss after THA. Although the direct participation of BMD is not clear, bisphosphonate was associated with a lower risk of aseptic revision in patients undergoing primary THA for osteoarthritis [6, 25]. Thus, the prevention of BMD loss by some anti-osteoporosis agents may be desirable in clinical setting. In fact, alendronate, a first-line drug for osteoporosis, was recognized as effective for the prevention of BMD loss around the implant [10, 11]. However, long-term continuous bisphosphonate treatment has been associated with atypical periprosthetic fractures [26, 27]. Periprosthetic fracture rates following primary THA were reported to be 1.1% in a large US cohort [28] and 0.64% at 10 years in the Swedish hip registry [29]. Moreover, bisphosphonate use was associated with a higher risk of periprosthetic fractures in younger patients with normal bone quantity [6]. Thus, an alternative drug may be desirable for the prevention of BMD loss after THA. A RCT showed that teriparatide and alendronate had equivalent effects [12]. However, patients on teriparatide require switching to another agent [30].

Although many previous studies showed that teriparatide was effective for osteoporosis treatment [31–36], the combination of parathyroid hormone (PTH) and alendronate was inferior to PTH alone in preventing loss of lumbar BMD [13]. In addition, a randomized trial found that switching from teriparatide to alendronate was superior to alendronate alone in maintaining spinal BMD [13]. Furthermore, teriparatide followed by alendronate resulted in greater gains in areal BMD than did alendronate alone at sites of rich cancellous bone [14]. Although the proportions of the cortical and cancellous bone at the femur have not been clearly determined and may depend on the individual, zone 1, in the greater trochanteric region, generally contains plenty of the cancellous bone. This may explain, at least, in part the results of the present study that there was no difference in ALD groups in zone 1. In this study, BMD loss in both zones 1 and 7 and the lumbar spine was significantly prevented in the switch group; therefore, switching teriparatide to alendronate was effective for periprothetic and lumbar spine. A major limitation was that the bone strength was not evaluated in this study. Biomechanical bone strength is not only dependent on BMD. Teriparatide has been reported to reduce peripheral or cortical volumetric BMD [33, 34, 37], although another study found that the femoral strength ratio was significantly higher in patients treated with teriparatide than with alendronate [38]. These results were consistent with findings showing that the correlation coefficient between change in femoral strength and change in trabecular volumetric BMD was higher in patients treated with teriparatide than with alendronate. In addition, teriparatide was reported to increase osseointegration and the mechanical strength of the bone-implant interface [39]. Thus, teriparatide may have several potential advantages regarding bone strength and/or increased osseointegration.

In conclusion, this randomized controlled trial assessed the effect of switching from teriparatide to alendronate in preventing BMD loss around the implant after THA. Switching therapy had a significant effect at the lumbar spine and the proximal femur around implants, including zones 1 and 7. BMD at zone 1 was significantly higher in the switch than in the control group, but no significant difference between ALD and control group. Switching from teriparatide to alendronate may be as useful as alendronate alone for preventing periprosthetic BMD after THA.

## Conclusions

Switching teriparatide to alendronate is more effective than alendronate alone in maintaining zone1 BMD and increasing lumber spine BMD.

## Data Availability

All data generated or analyzed during this study are included in this published article [and its supplementary information files].

## Abbreviations

ALD: Alendronate
AP: Anterior-posterior
BMD: Bone mineral density
DEXA: Dual-energy X-ray absorptiometry
HHS: Harris Hip Score
JOA: Japanese Orthopaedic Association
PTH: Parathyroid hormone
RCT: Randomized controlled trial
ROIs: Regions of interest
THA: Total hip arthroplasty
UCLA: University of California, Los Angeles
UMIN: University Hospital Medical Information Network
